# How to Modulate Tumor Hypoxia for Preclinical In Vivo Imaging Research

**DOI:** 10.1155/2018/4608186

**Published:** 2018-10-18

**Authors:** Sven De Bruycker, Christel Vangestel, Steven Staelens, Tim Van den Wyngaert, Sigrid Stroobants

**Affiliations:** ^1^Molecular Imaging Center Antwerp (MICA), University of Antwerp, Universiteitsplein 1, 2610 Wilrijk, Antwerp, Belgium; ^2^Department of Nuclear Medicine, Antwerp University Hospital (UZA), Wilrijkstraat 10, 2650 Edegem, Antwerp, Belgium

## Abstract

Tumor hypoxia is related with tumor aggressiveness, chemo- and radiotherapy resistance, and thus a poor clinical outcome. Therefore, over the past decades, every effort has been made to develop strategies to battle the negative prognostic influence of tumor hypoxia. For appropriate patient selection and follow-up, noninvasive imaging biomarkers such as positron emission tomography (PET) radiolabeled ligands are unprecedentedly needed. Importantly, before being able to implement these new therapies and potential biomarkers into the clinical setting, preclinical in vivo validation in adequate animal models is indispensable. In this review, we provide an overview of the different attempts that have been made to create differential hypoxic in vivo cancer models with a particular focus on their applicability in PET imaging studies.

## 1. Background

**Figure 1:**
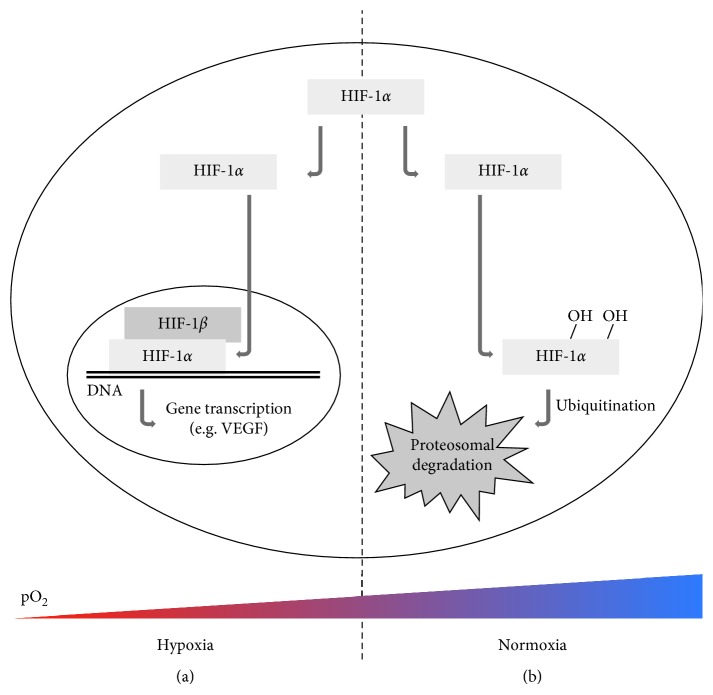
The HIF-1 pathway. (a) Under hypoxic conditions, HIF-1*α* stabilizes, and its transcriptional activity is regulated by heterodimerization with the constitutively expressed HIF-1*β* and binding to the hypoxia-response elements in the promoters of genes that regulate a variety of biological processes. These include genes involved in tumor survival, progression, and proliferation, such as VEGF. (b) Under normoxic conditions, the HIF-1*α* subunit is rapidly degraded by the proteasome after hydroxylation and ubiquitination.

Hypoxia, which frequently occurs in solid tumors, is related with an aggressive phenotype, chemo- and radiotherapy resistance, and thus a poor clinical outcome. To a considerable extent, hypoxia-inducible factor-1 (HIF-1), the major transcriptional regulator of the cellular response to hypoxia (Figure 1), is responsible for these observed phenomena. Encouragingly, tremendous progress with strategies to overcome the unfavorable effects of hypoxia has been made, paving the way for new personalized medicine opportunities. However, as not all patients will benefit from this new, promising radiosensitizing treatment schedules, molecular biomarkers are of utmost importance for adequate patient selection. Imaging biomarkers, such as positron emission tomography (PET) radiolabeled compounds, allow noninvasive and longitudinal assessment of molecular and functional characteristics of a tumor, thereby coping with inter- and intratumoral heterogeneity. Therefore, over the past decades, imaging biomarkers have tremendously increased in significance.

From this perspective, adequate animal models may accelerate translation of new therapies and personalized approaches, and also potential new molecular imaging biomarkers, into routine clinical practice. In hypoxia research in particular, it is often crucial to be able to distinguish between a positive and negative model for tumor hypoxia, i.e., tumors that are poorly and well oxygenated, respectively. Obviously, these models should also accurately reflect human disease. From this point of view, cell-line derived xenograft models may be inferior to genetically engineered models, orthotopic models, or patient-derived xenografts [1]. However, based on historic knowledge obtained in subcutaneous xenograft models, the fact that tumors are externally visible and easily measurable and the relatively straightforward experimental procedures that are required for their creation and follow-up, subcutaneous models are still frequently used in preclinical cancer studies, including hypoxia research.

A prerequisite to be able to differentiate between a positive and negative model for tumor hypoxia is the availability of methods to quantify tumor oxygenation. To date, a variety of techniques has been used to measure tumor hypoxia, as recently reviewed by Fleming et al. [2]: oxygen (O_2_) electrodes such as OxyLite; electron paramagnetic resonance (EPR); histological assessment with extrinsic biomarkers, mainly pimonidazole, and intrinsic biomarkers such as carbonic anhydrase IX (CAIX); blood O_2_ level-dependent (BOLD) magnetic resonance imaging (MRI); and single photon emission tomography (SPECT) or PET with for instance hypoxia-targeting 2-nitroimidazole-based radiotracers [2, 3]. However, correlations between the different measuring techniques are often absent [4, 5] as they all provide information on different locations within the tumor, e.g., intracellular hypoxia, interstitial hypoxia, or blood oxygenation [2]. Moreover, some techniques such as OxyLite inevitably damage the tumor tissue. Therefore, the question remains if one of these techniques can be considered as the reference standard for measuring tumor hypoxia.

**Figure 2:**
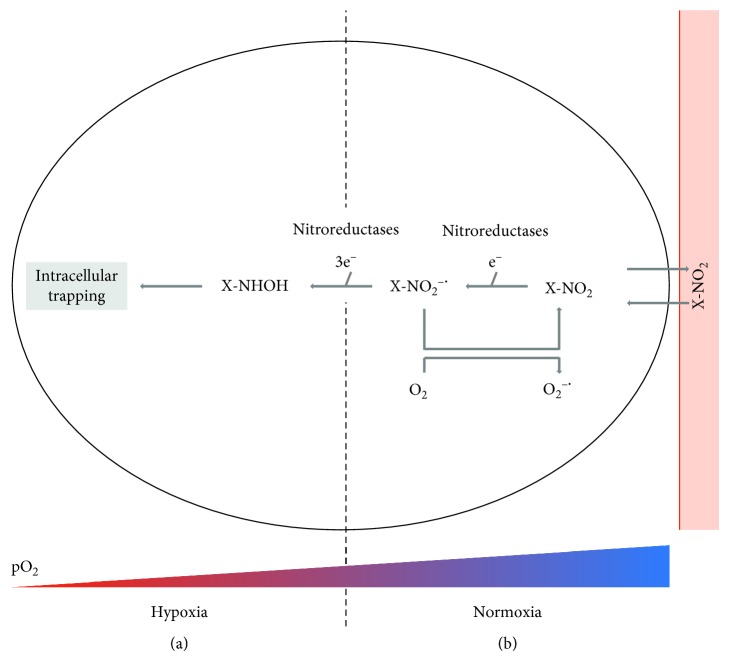
The binding mechanism of 2-nitroimidazole tracers here represented as X-NO_2_. (a) Under hypoxic conditions, tracer diffuses into the cell, where the NO_2_ group undergoes a series of reductions and some of the intermediate products that are formed during these reactions will bind to macromolecules within the cell. (b) Under normoxic conditions, the first reduction is reversed, giving rise to the original compound which can unhinderedly exit the cell. Importantly, for removal of 2-nitroimodazole background signal, an uptake time of minimal 3 hours is needed [3].

Over the past decades, several 2-nitroimidazole derivatives have been developed for hypoxia PET imaging (Figure 2). [^18^F]fluoromisonidazole ([^18^F]FMISO), considered as the prototype tracer, is a lipophilic compound that is highly metabolized in the liver and cleared via hepatobilary and gastrointestinal pathways. The more hydrophilic next-generation 2-nitroimidazole derivatives, such as [^18^F]flortanidazole ([^18^F]HX4), have better pharmacokinetic properties, resulting in chiefly renal excretion of the intact compound and only limited (<20%) hepatobilary clearance. This gives rise to improved hypoxia-to-normoxia tissue ratios and thus images with higher contrast in comparison to [^18^F]FMISO [2,6–9].

Molecular imaging techniques, such as PET with 2-nitroimidazole-based probes (Figure 2), may offer considerable advantages over the other techniques because of their noninvasive nature, accuracy and reliability, and the opportunity to measure hypoxia directly, both spatially and temporally [2, 10]. From the preclinical point of view, PET imaging has the added advantages that its use is directly translatable to the clinic and that the same set of animals can be followed over time, thereby reducing the required number of animals for a single experiment and allowing individual therapy response assessments. Since animals function as their own controls over time, their intrinsic intra-animal analysis increases statistical power [1].

Furthermore, PET imaging allows absolute quantification of hypoxia. Ideally, this is achieved by performing dynamic acquisitions and kinetic modelling [11]. Accordingly, longer anesthesia regimens will be required, and especially for hypoxia imaging, this may induce (re)oxygenation (cf Section 2.3.1). Evidently, dynamic imaging also decreases the experimental throughput [12] which might prove cumbersome in large groups of animals ideally being scanned in identical hypoxia settings. Therefore, most studies use semiquantitative parameters derived from static images, such as standardized uptake values (SUV) or tumor-to-background ratios (TBR). Importantly, as it has been shown that these static parameters are time-dependent [3, 13, 14], it should always be taken into account that these semiquantitative approaches inherently lead to higher degrees of inaccuracy. To a certain extent, this can be overcome by respecting and maintaining an invariable tracer uptake time of at least 3 hours within an experimental set-up [3, 13]. However, for the more hydrophilic next-generation hypoxia tracers such as [^18^F]HX4, which are dependent on renal clearance, interanimal variability will remain substantial, due to intrinsic differences in kidney function [3]. In line with this, in therapy response evaluation studies, in which animals undergo multiple PET scans (i.e., pre- and post-therapy), drug-induced alterations of renal excretion may influence tracer clearance [3]. Unfortunately, no established standardized quantification methodologies have been developed for hypoxia PET imaging yet, particularly in the preclinical setting. Therefore, the difficulties discussed above need to be confronted when analyzing hypoxia PET images.

**Figure 3:**
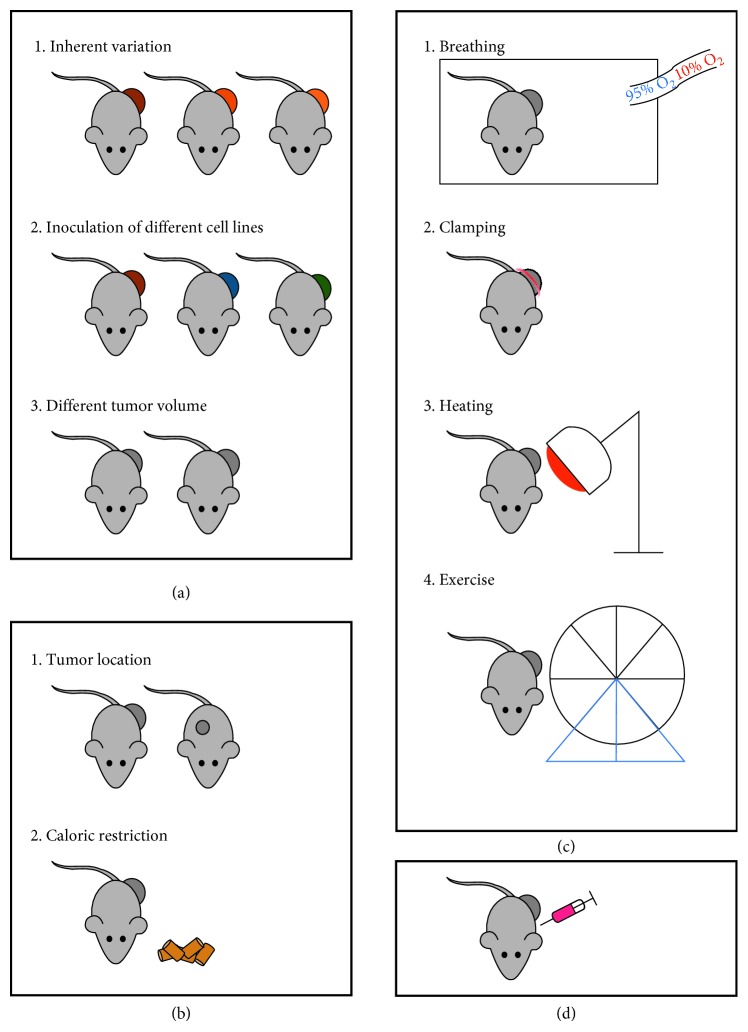
In vivo hypoxia modulation. Overview of the different experimental techniques and study designs that may be applicable for manipulating the tumor oxygenation state for hypoxia research. (a) Tumor physiological parameters. (b) Animal physiological parameters. (c) External interventions. (d) Pharmacological interventions.

In this review, we provide an overview of the different experimental approaches and study designs that may be applicable for manipulating the tumor oxygenation state for in vivo hypoxia research (Figure 3) with a particular focus on preclinical hypoxia PET imaging, considering the inherent difficulties of this imaging technique. For each modulation approach, we will indicate its respective opportunities and pitfalls, and share our own experiences and the difficulties we run up against in our own attempts to create a differential hypoxia murine cancer or tumor model.

## 2. Generating Differential Hypoxia in Tumor Models

### 2.1. Tumor Physiological Parameters

#### 2.1.1. Inherent Variation without Manipulations

The ability to detect differential hypoxia in a single xenograft model without underlying external manipulation is the ultimate paradigm for in vivo cancer hypoxia research. However, where inoculation of some cancer cell lines inherently leads to tumors with different degrees of tumor hypoxia regardless of the tumor volume [15], this is not the case for other cell lines. For instance, heterogeneous [^18^F]-2-nitroimidazol-pentafluoropropyl acetamide ([^18^F]EF5) uptake was observed in A549 non-small cell lung cancer (NSCLC) xenografts, but not in RKO and HT29 colon carcinoma xenograft models [16].

In some xenograft models, the observed intertumoral variation in tumor oxygenation allowed to show the properties of hypoxia PET as a predictive biomarker. For instance, considerable intertumoral variation in oxygenation has been observed in 9L glioma tumors, which enabled the detection of significant correlations between [^18^F]EF5 uptake and response to single-dose radiotherapy [17]. In line with these results, Beck et al. performed [^18^F]fluoroazomycin arabinoside ([^18^F]FAZA) imaging on 67 EMT6 breast tumor-bearing mice and used the median tumor-to-background ratio to discriminate between hypoxic and normoxic tumors. Based on this distinction, significantly faster tumor growth of the hypoxic tumors as compared to their normoxic counterparts was observed. Moreover, it was possible to show that administration of the radiosensitizer tirapazamine prior to radiotherapy was beneficial in hypoxic, but not in normoxic, tumors. Importantly, in this study, tumor volumes were uniformly distributed across both groups [18]. Similarly, it was found that baseline [^18^F]FAZA TBR, which ranged from 1.17 to 5.83, was higher in radioresistant than in radiosensitive esophageal carcinoma xenograft tumors [19]. Finally, [^18^F]FAZA TBR predicted that rhabdomyosarcoma and glioma tumors were sensitized to the effects of radiotherapy by nimorazole [20] and identified colorectal xenograft tumors that benefited from addition of the hypoxic prodrug evofosfamide to standard chemoradiotherapy regimens [21].

Despite these promising results which have been successful in validating the predictive character of hypoxia PET, this approach is not without pitfalls. For instance, in most cases, rather large cohorts of animals may be required to detect differential hypoxia, which is not preferable from an ethical point of view. Also, the relationship between tumor volume and oxygenation as such can also be an interfering factor and will therefore be discussed in a separate section (2.1.3) within this review. The most important concern may however be the lack of a clear cutoff value to discriminate between “hypoxic” and “normoxic” (i.e., “less hypoxic”) tumors in these studies. Indeed, the chosen thresholds that have been reported seem rather arbitrary [4,16,18–20,22], which renders the comparison of different studies difficult. Also, interpretation of intermediate values, i.e., values fluctuating around the threshold, may be complicated. Moreover, as these studies are predominantly semiquantitative and both SUV and TBR are extremely dependent on the pharmacokinetic properties of the used 2-nitroimidazole tracer and the PET imaging protocol [3, 14, 23], the comparison of studies with different tracers may be impossible in any case.

#### 2.1.2. Inoculation of Different Cell Lines

The unique characteristics of different cancer cell lines give rise to different tumor phenotypes with typical features that will conceivably lead to differential tumor hypoxia. Using pimonidazole immunostaining and [^18^F]FMISO, [^18^F]-2-nitroimidazol-trifluoropropyl acetamide ([^18^F]EF3) and [^18^F]EF5 PET, differential tumor hypoxia could be observed in animal models of osteosarcoma [24], fibrosarcoma [7], glioma [25–27], head and neck cancer [28–31], lung cancer [15], melanoma [32], and prostate cancer [15, 33, 34] that were all derived from different cell lines, and in a panel of colorectal cancer (CRC) patient-derived xenograft tumors [21]. Similarly, the different stages of castration responsiveness in the Shionogi prostate cancer tumor model could be differentiated using [^18^F]EF5 PET [35].

The investigation of different cell lines may be very relevant from a clinical point of view, as it may accurately reflect the high degree of intra- and intertumor heterogeneity observed in and between cancer patients. However, for fundamental research purposes, this approach may result in very complex analyses. When for instance therapy response is compared between different models, the different oxygenation status of the tumors may be only one of many factors that influence therapy outcome. Indeed, the diverse genetic and phenotypic profiles arising from the different cell lines will also contribute to therapy responsiveness independently from the degree of hypoxia.

To undo the potential complexity of comparing experimental results obtained in tumors arising from different cell lines, knockdown or knockout cell lines can be used. In these cells, the expression of only one or some genes is either reduced or prevented, respectively. This allows the investigation of the functional roles of particular genes. The most obvious gene to be eliminated in regard to tumor hypoxia is HIF-1 (Figure 1). Using this approach, an inhibition in tumor growth was observed in subcutaneous HIF-1 knockout HCT116, but not RKO CRC xenograft models. Interestingly, the amount of hypoxia as determined with pimonidazole was not affected [36]. In subcutaneous lung and gastric xenograft tumors on the other hand, HIF-1*α* knockdown stimulated tumor growth [37–39]. In the case of gastric cancer, knockdown additionally resulted in aggressive peritoneal dissemination via upregulation of matrix metallopeptidase-1 and in increased sensitivity to 5-FU chemotherapy through increased susceptibility to apoptosis and downregulation of drug efflux transporters [37–39]. In subcutaneous xenografts derived from HIF-1*α* deficient embryonic stem cells, HIF-1*α* deficiency resulted in accelerated tumor growth, decreased perfusion, and increases in tumor hypoxia as observed by pimonidazole staining [40, 41]. Genetic ablation of vascular endothelial growth factor (VEGF), a proangiogenic signal protein that is one of the downstream targets of HIF-1 (Figure 1), has been shown to give rise to increases in pimonidazole staining and thus tumor hypoxia on top of reductions in vascularity and tumor volume in a variety of tumors [36, 41–43]. Interestingly, genetic disruption of both HIF-1 and VEGF further inhibited CRC xenograft tumor growth as compared to VEGF disruption alone, but no additive effect on the hypoxic tumor compartments could be observed [36].

It should however be noted that the observed alterations in tumor vasculature rather than the degree of hypoxia itself may have influenced pimonidazole uptake (cf Section 2.4). Logically, this potential complication factor may also hold in PET imaging studies. The observed effects may also depend on the inoculation site. For instance, subcutaneous inoculation of HIF-1*α*-deficient and VEGF-deficient transformed astrocytes resulted in reduced vessel density and tumor growth. Intracranial inoculation on the other hand led to accelerated growth of HIF-1*α*-deficient tumor growth, whereas VEGF-deficient astrocytomas still exhibited a growth disadvantage. This suggests that the differences in the microenvironment and the vascular structure between the two inoculation sites determine the behavior and aggressiveness of the tumor [44]. The influence of the inoculation site on tumor hypoxia will be discussed more extensively in Section 2.2.1.

**Figure 4:**
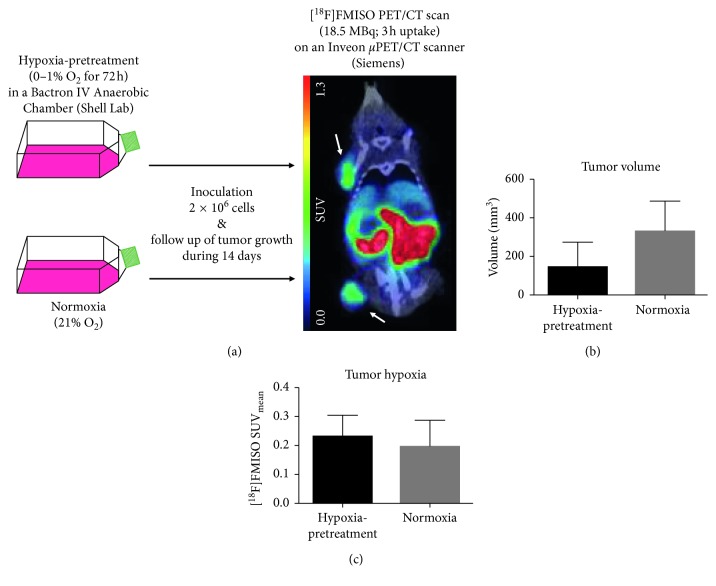
Hypoxia-pretreatment experiment, approved by the Antwerp University Ethical Committee (2012-69). All applicable institutional and European guidelines for animal care and use were followed. (a) Colo205 cells (Perkin Elmer) were exposed to hypoxia (0-1% O_2_) for 72 h in a Bactron IV Anaerobic Chamber (Shell Lab) or to normoxia (21% O_2_) in a common incubator. CD-1 nude mice (Charles River, *n*=5) were inoculated with 2 × 10^6^ “hypoxic” cells in the shoulder and with 2 × 10^6^ “normoxic” cells in the hind leg. Fourteen days later, animals underwent a [^18^F]FMISO PET/CT scan as previously described [53]. Images were analyzed as previously described [53]. One representative mouse is shown. Arrows indicate tumors. As [^18^F]FMISO clearance mainly occurs via the hepatobilary pathway and the gastrointestinal tract [8], tracer uptake can be observed in liver and intestines. (b) “Hypoxic” tumors were numerically, but not significantly, smaller than “normoxic” tumors (144 ± 129 mm^3^ vs 328 ± 158 mm^3^ [mean ± SEM], respectively; *p*=0.19). (c) [^18^F]FMISO SUV did not differ between “hypoxic” and “normoxic” tumors (0.228 ± 0.074 vs 0.195 ± 0.090, respectively; *p*=0.71).

To wind up with, in orthotopic MDA-MB-231 breast cancer xenograft models, exposure of cells to hypoxic culturing conditions prior to inoculation not only accelerated tumor growth, but also contributed to multidrug resistance, most importantly via increased HIF-1*α* levels. Indeed, the hypoxia-driven induction of a differential protein expression makes this technique very interesting for imaging purposes. However, differences between tumors derived from hypoxic and normoxic cells tend to diminish with increases in tumor volume, starting from 100 mm^3^ onwards [45]. Moreover, the effects of in vitro exposure of cells to hypoxic conditions prior to inoculation may be tissue-dependent or cell line-dependent. Indeed, in recent studies, it was shown that xenografts from lung cancer cells cultured under hypoxic conditions show decelerated tumor growth but enhanced cell survival, whereas the same strategy resulted in accelerated subcutaneous tumor growth in a CRC model [46, 47]. In our own quest to create a differential hypoxic xenograft model, we investigated the effect of hypoxia-pretreatment of Colo205 cells 72 hours prior to inoculation. Fourteen days after inoculation, we observed a nonsignificant Colo205 tumor growth inhibition of 56% of the tumors arising from hypoxia-pretreated cells, but [^18^F]FMISO uptake was not affected by the hypoxia-pretreatment (Figure 4). This may not be surprising since tumor volume may also affect hypoxia tracer uptake, as discussed in Section 2.1.3. Therefore, seeing the unpredictable effect on tumor proliferation, we do not believe that exposure of cells to hypoxic conditions prior to inoculation is a reliable method for in vivo tumor hypoxia modulation.

#### 2.1.3. Tumor Volume

In rat rhabdomyosarcoma tumors, it was observed that the hypoxic volume assessed with both pimonidazole staining and [^18^F]FMISO autoradiography increased with increases in tumor volume [48]. In murine sarcoma models on the other hand, inverse correlations between autoradiographically determined [^18^F]fluoroerythronitroimidazole ([^18^F]FETNIM) and tumor volume were found [49], whereas in other models, [^18^F]FETNIM, [^18^F]FMISO, or [^18^F]FAZA uptake did not correlate with tumor volume [50–52]. These conflicting observations may be due to the presence of tumor necrosis [49–51], since, 2-nitroimidazole PET tracers will be not retained by necrotic cancer cells. As necrosis may be more wide-spread in larger tumors [16], whether or not areas of necrosis are included within the determined volume of interest (VOI) can potentially lead to underestimation or slight overestimation of the amount of tumor hypoxia, respectively, dependent on the quantification method. These important observations should be considered particularly when exclusively focusing on tumor volume.

The use of autoradiographs allows an easy and accurate quantification of the amount of tumor necrosis, although only in ex vivo tissue samples. Corrections for the influence of necrosis on in vivo hypoxia are more complicated. For instance, in a CH3 mammary carcinoma model, [^18^F]FMISO TBR did not correlate with tumor volume, despite the existing correlation between tumor volume and pO_2_ electrode measurements [5]. Again, this apparent discrepancy between these two measuring techniques has been attributed to the presence of tumor necrosis [54]. Indeed, the automatic thresholding technique which was used in this study to delineate tumors on PET images [5] may exclude macroscopically necrotic areas without tracer uptake. The polarographic method on the other hand cannot distinguish between viable and necrotic tumor fractions. Interestingly however, by applying necrosis correction in murine rhabdomyosarcoma tumors, it was observed that polarographically-determined pO_2_ values did not really change as tumors grew larger and did not correlate with the degree of necrosis, unless when tumors reached a weight of more than 2 grams [55].

Depending on the tumor delineation method (manual or automatic) on static PET images, areas of macroscopic necrosis will or will be not included within the VOI. Importantly, in studies that investigated the relationship between tumor volume and hypoxia PET tracer uptake [16,23,26,27,31,33,56–58], it is not always clear if areas of necrosis were included within the VOI, which renders the interpretation and comparability difficult. Moreover, the resolution of preclinical PET may in any case be too low to discriminate areas of microscopic necrosis. The use of parametric images based on different tracer uptake patterns over time between normoxic, hypoxic, and necrotic regions also looked promising to overcome these shortcomings [11], but the need for dynamic imaging may preclude general use in rodent models for tumor hypoxia.

To what extent tumor volume influences the degree of hypoxia remains an intriguing question. Therefore, we do not recommend the use of different volumes as a model for tumor hypoxia for preclinical PET investigations. If opting for this methodology regardless, the exclusion of macroscopic areas of necrosis during hypoxia PET tracer quantification is advisable.

Taken together, to be able to make a more well-defined distinction between “hypoxic” and “normoxic” tumors, and to broaden the intertumoral oxygenation range, experimental manipulation may be required. In what follows we will discuss the different procedures that have been adopted for this purpose.

### 2.2. Animal Physiological Parameters

#### 2.2.1. Tumor Location

In a study by Graves et al., the uptake of the glucose analogue 2-deoxy-2-[^18^F]fluoro-D-glucose ([^18^F]FDG), [^18^F]FAZA, and pimonidazole was compared between genetically induced lung tumors in in situ, subcutaneous, and orthotopic A549 xenograft models. [^18^F]FDG uptake was comparable between all models, whereas [^18^F]FAZA and pimonidazole were only trapped in subcutaneous tumors, but not in lesions growing within the lung. Those observations were confirmed by administering the hypoxic prodrug PR-104 to all models, as therapy response was only observed in subcutaneous tumors [59]. Taken together, these data show that the presence of hypoxia in lung cancer may depend on the inoculation site and thus the present microenvironment. It has been hypothesized that this may be either a result of the tumor vasculature being more functional in orthotopic than in subcutaneous tumors, or the accessibility of O_2_ via the alveoli in orthotopic but not in subcutaneous tumors, thereby suggesting that this inoculation site-specific differential hypoxia is seen exclusively in lung cancer [60]. Nevertheless, our research group recently observed significantly lower tumor hypoxia, as assessed with CAIX immunohistochemistry, in an orthotopic CRC xenograft model as compared to subcutaneous tumors [61].

As already mentioned earlier in Section 2.1.2, the growth and differentiation of HIF-1*α*-deficient and VEGF-deficient astrocytomas is also dependent on the inoculation site and its corresponding microenvironment [44]; however, the degree of hypoxia was not quantified in the cited study. Similarly, it has been observed that orthotopically and subcutaneously grown gliomas derived from the same cell line exhibit different gene expression profiles [62]. Thus, it is clear that the inoculation site influences tumor characteristics. Again, a major drawback of such technique is the different growth rate of subcutaneously and orthotopically growing tumors. Moreover, the different growth patterns [59] will inevitably lead to different tumor volumes. Both factors may complicate the interpretation of data on tumor hypoxia (cf Section 2.1.3).

#### 2.2.2. Caloric Restriction

The Warburg effect, i.e., the upregulation of glycolysis in cancer cells regardless of the partial O_2_ pressure, accounts for tumors' high dependency on the supply of nutrients, especially glucose. Therefore, caloric restriction (CR), which is the reduction in energy uptake without malnutrition as compared to ad libitum feeding, has been considered as a promising synergistic treatment option. CR can be achieved via different ways: via intermittent fasting, via short-term fasting, or via chronic daily energy restriction whereby animals are only fed a certain percentage of normal intake [63]. As tumor hypoxia is inextricably bound with the Warburg effect, CR may provide another obvious way to experimentally influence tumor oxygenation. Indeed, in A549 lung cancer xenograft models, it was shown with EF5 immunostaining that daily food restriction caused significant decreases in tumor hypoxia, on top of significant inhibition of tumor growth. In line with this, HIF-1*α* expression, VEGF expression, and microvessel density (MVD) were all reduced [64]. Similar observations were made in rat, mouse, and human brain tumor models, where decreases in HIF-1*α*, VEGF, and MVD could be observed [65, 66]. In line with this, it was shown in orthotopic allograft breast cancer models that CR and radiotherapy worked synergistically [67]. Within the context of tumor hypoxia, VEGF inhibition by CR was suggested to account for its observed radiosensitizing capacities, as this may result in vascular normalization and thus increases in the O_2_ tension [63].

Taken together, these data are promising for the use of CR to create a differential hypoxic tumor model. However, it has been reported that some cell lines may be resistant for CR [68]. Moreover, some considerations should be taken into account when using CR in combination with PET imaging. First, CR lowers blood glucose and body weight [63–67] and these disturbances should be taken into account during PET image quantification. Second, the dietary status may determine the intratumoral tracer distribution pattern. In A549 xenograft models, [^18^F]FDG accumulated predominantly in the hypoxic cancer cells of fasted animals, whereas in fed animals, radiotracer accumulated in the noncancerous stroma [69]. Third, CR also influences tumor volume [64–67], which potentially could alter tumor hypoxia as discussed in Section 2.1.3.

### 2.3. External Interventions

#### 2.3.1. Breathing

Mortensen et al. separated a hypoxic and a normoxic mammary carcinoma group based on the median [^18^F]FAZA TBR, although in this study, intertumoral oxygenation heterogeneity was increased by exposing some of the animals to carbogen breathing prior to [^18^F]FAZA scanning and radiotherapy [4]. The exposure of tumor-bearing animals acutely or chronically to a gas mixture containing a reduced O_2_ concentration (e.g., 5–10% O_2_) or, exactly the opposite, to an increased O_2_ concentration (most commonly carbogen, i.e., 95% O_2_), whether or not under hyperbaric pressure conditions, is indeed the most applied technique to modulate tumor oxygenation in vivo. In this way, O_2_ diffusion distances decrease or increase, respectively, thereby deteriorating or improving the degree of chronic hypoxia within the tumor. In the study of Mortensen et al., significantly different tumor control probabilities were observed in both tumor groups. Interestingly, when the carbogen-breathing animals were excluded from the analysis, significance of the radiotherapy efficacy was lost. The authors therefore correctly suggested that in general, intertumoral variability in tumor hypoxia within one model may be too low without any external manipulations [4].

In a variety of cancer xenograft models, the breathing technique has proven to be very useful to detect different degrees of hypoxia with numerous hypoxia PET tracers, including [^18^F]FMISO [5, 6, 14, 29, 50, 53, 70–72], [^18^F]EF3 [6, 7, 73], [^18^F]FETNIM [50], [^18^F]FAZA [4, 14, 22, 71, 74, 75], [^18^F]fluoroetanidazole ([^18^F]FETA) [76], and [^18^F]HX4 [8, 14, 77]. An important issue of this breathing technique is that tracer metabolism and trace uptake of background regions may also be affected [14], which may hamper image quantification using background normalization. It has been observed that carbogen or hypoxic gas breathing significantly altered [^18^F]FMISO uptake in fat tissue [50] and [^18^F]FETNIM and [^18^F]FAZA uptake in muscle [14, 50, 71]. Similar observations were made by Cairns et al., who found drops in pO_2_ in normal muscle tissue when animals breathed 5–7% O_2_ and rises in pO_2_ when ambient air was reintroduced [78]. Taken together, exposure to an altered breathing atmosphere obviously alters hypoxia tracer uptake of noncancerous tissue too. Therefore, it may not be appropriate to calculate TBRs, without thorough understanding of the magnitude and dynamics of these changes. By contrast, it has also been argued that calculating TBRs is a way to anticipate the possible confounding effects on background tracer uptake [75].

Carbogen breathing is often combined with the administration of nicotinamide, a vitamin B3 derivate that induces vasodilation and thus improves tumor perfusion. In this way, not only chronic but also acute hypoxia can be counterbalanced [79]. Indeed, regional short-lived changes in blood flow could possibly influence tracer circulation and cause considerable day-to-day variations in hypoxia tracer uptake. Nevertheless, given the relatively low temporal resolution of (micro)PET (i.e., 10 seconds dynamic frames) and the long uptake period required for 2-nitroimidazole-based tracers, acute hypoxia may not be detectable with PET [80]. Moreover, in different models, it was shown that the combination of carbogen and nicotinamide gave rise to similar or even less pronounced decreases in the hypoxic fraction compared to carbogen alone [81, 82]. These two facts question the additional value of nicotinamide. In spite of this, it has been observed that depending on the amount of carbon dioxide in the gas mixture, breathing of a high-O_2_ gas mixture may cause vasoconstriction [83], thereby deteriorating tumor oxygenation state and also tracer delivery. In this particular case, administering nicotinamide may compensate for that [75, 81, 84], but it should be noted that changes in tumor vascularity are not necessarily accompanied by a reduced accessibility of radionuclide tracers to tumor tissue [85, 86]. The eventual positive or negative influence of breathing an altered atmosphere on the oxygenation state and on tumor progression appears to be tissue-dependent and cell line-dependent [46, 83, 87–89].

The effectiveness of breathing models has also been shown in numerous experiments in which hypoxia-evoked therapy resistance was investigated. The most straightforward, yet clinically less relevant approach is the investigation of the influence of breathing an altered O_2_ atmosphere on the effect of a single therapy dose. For instance, in fibrosarcoma, colon carcinoma, and hepatoma models, tumor growth decreased significantly when a single dose of 5-fluorouracil (5-FU) was administered during short-term carbogen breathing [90–93]. In line with these results, our study demonstrated a reduced 5-FU chemotherapy effect in colorectal carcinoma xenografts exposed to short-term hypoxic breathing conditions during administration of a single dose of 5-FU, as predicted on a baseline [^18^F]FMISO scan [53]. In yet another study, rhabdosarcoma-bearing rats and lung tumor-bearing mice were exposed to modified O_2_ concentrations 4 hours per day during 5 consecutive days. Using this approach, the investigators were able to show that treatment efficacy of the hypoxic prodrug TH-302 was dependent on tumor oxygenation: daily short-term exposure of the xenograft models to carbogen abolished the effect of TH-302, whereas 7% O_2_ breathing increased the therapeutic potential [77]. Accordingly, the hypoxic prodrug CEN-209 was induced more effectively in subcutaneous HCT116 tumors of mice exposed to a hypoxic atmosphere, whereas hyperbaric O_2_ breathing suppressed CEN-209 activation when the drug was administered after irradiation [94]. The use of breathing an altered atmosphere for improving radiotherapy response is also being studied extensively. For instance, accelerated radiotherapy with carbogen and nicotinamide (ARCON) enhanced therapeutic response in a variety of tumor models, including mammary adenocarcinoma and sarcoma models [88, 95, 96].

Nevertheless, the breathing approach may become technically challenging for longitudinal therapy schedules. In particular, it has been shown in murine and rat tumors that the effect of breathing an adapted atmosphere can be reversed immediately when the intervention stops [82, 97, 98], is time-dependent [84], and may even be lost after prolonged exposure times [22, 99]. Things may even become more complex when considering that anesthesia potentially influences the experimental outcome. Indeed, to avoid motion-related artefacts during preclinical in vivo imaging, the use of anesthetics is inevitable, and these anesthetics or their carrier gasses (in case of volatile drugs) can have a substantial influence on tumor oxygenation. For instance, drops in pO_2_ in tumor and muscle tissue of ketamine/xylazine-anesthetized animals when compared to isoflurane-anesthetized mice have been observed [100]. In CT26 colorectal carcinoma-bearing mice, [^18^F]FAZA PET uptake was increased in both tumor and muscle tissue in ketamine/xylazine-anesthetized animals as compared to isoflurane-anesthetized animals. Yet these changes were less pronounced in muscle, resulting in higher [^18^F]FAZA TBR in ketamine/xylazine-anesthetized animals [101]. In a murine adenocarcinoma model on the other hand, no difference in [^18^F]FMISO TBR between the two anesthesia regimens was found, despite the observation of higher tumoral uptake in ketamine/xylazine-anesthetized animals compared to their isoflurane counterparts [102]. Surprisingly however, in both imaging studies, it was shown that [^18^F]FAZA and [^18^F]FMISO TBR, respectively, did not differ between animals breathing room air or animals breathing O_2_ during isoflurane anesthesia [101, 102].

#### 2.3.2. Clamping

The O_2_ state of tumors can be temporarily reduced by physically occluding the blood supply to the tumor. This clamping technique gives rise to severe hypoxia. In general, animals are subcutaneously inoculated in the hind leg. When tumors have reached the desired volume, animals are anesthetized whereupon the proximal part of the leg is clamped with a rubber band or a metal clamp for a limited time period. In this way, transient acute tumor hypoxia is mimicked. For instance, it was observed that clamping induces expression of HIF-1*α* [103]. Deteriorated radiotherapy response after clamping has also been observed [104]. Similarly, this clamping technique has been successfully applied in an orthotopic liver tumor model, where severe hypoxia could be induced via hepatic artery ligation [105].

On top of being painful and stressful [106], clamping has some other major limitations. First, by occluding the blood supply to the tumor, the delivery of therapy or radiotracer will also be extremely hampered [104]. It was hypothesized that this could be prevented by administering [^18^F]FMISO prior to initiation of the clamping procedure. Indeed, in two different glioblastoma models, this approach resulted in significantly increased tracer and pimonidazole uptake. On the opposite, it was emphasized that clamping after PET tracer administration may just cause trapping of unbound radiotracer in the tumor [107]. A second drawback of the clamping technique is that ischemia can occur instead of hypoxia, leading to irreversible damage and cell death. Third, the occluded artery may clot during the clamping, hampering reperfusion [105].

#### 2.3.3. Temperature Modification

As excellently reviewed by Song et al. [108], ample evidence in a variety of xenograft models shows that tumor oxygenation improves during and after heating at 39 to 42°C. This observation is probably the result of a combination of an increase in tumor blood perfusion and a decrease in the O_2_ consumption rate. Moreover, mild hyperthermia oxygenates both chronic and acute hypoxic cells, and this effect may last for up to 48 hours after heating [108]. A second proposed mechanism of action of hyperthermia is that it may kill hypoxic cells directly [109, 110]. In order to avoid this so-called hyperthermic cytotoxicity, which is beneficial from the therapeutic point of view, but may complicate therapy response evaluation experiments focusing on tumor hypoxia, the applicable temperature range is rather narrow.

Practically, the tumor-bearing leg of restrained or anesthetized animals is immersed into heated water. In a subcutaneous CRC model, this resulted in an immediate decrease in the hypoxic fraction, assessed with the extrinsic hypoxia markers pimonidazole and EF5, but the effect disappeared 24 hours after heating [111]. In a mammary carcinoma model, HIF-1*α* increased immediately after 1 hour of hyperthermia treatment and was restored 48 hours later. VEGF however was elevated up to 48 hours after treatment, which on its turn was responsible for the induction of angiogenesis, increased tumor perfusion, and consequent decreases in tumor hypoxia [112].

Another way to achieve hyperthermia is whole-body heating. By placing mice in a heated environmental chamber, body temperature and thus tumor temperature can be raised, leading to improved tumor perfusion [113]. After 6 hours of whole-body heating, pimonidazole staining showed an initial decrease in hypoxia in a CRC xenograft model, possibly by an increase in vascular volume, but slightly increased again after heating [113].

Although it was suggested that temporal increases in HIF-1*α* may lead to tumor reoxygenation and may therefore be beneficial in particular cases [112], the role of HIF-1 in therapy resistance is irrefutable and its upregulation via heating may most likely be detrimental. Second, raising the temperature of deep-seated orthotopic tumors may be technically challenging or even impossible using external heating. Whole-body heating may overcome this short-coming but can cause systemic stress. Third, it has been argued that anesthetics, which are indispensable in imaging research, may limit the thermoregulatory response as they cause vasoconstriction and thus may hamper the efficacy of hyperthermia [113]. Fourth, seeing its transient nature, long-term use of hyperthermia may be complicated. Thus, tumor heating may not be a straightforward approach for the creation of a differential hypoxic tumor model.

#### 2.3.4. Exercise

In two studies by Jones et al., the effects of long-term voluntary wheel running on tumor perfusion, hypoxia, and angiogenesis were investigated in two different orthotopic cancer models. In both models, significantly higher hypoxia, epitomized by an increase in HIF-1*α* expression, and also improved tumoral blood perfusion were observed in the exercise group as compared to the sedimentary group. As speculated by the authors, exercise may cause an increase in O_2_ delivery towards muscle and heart tissue, thereby redirecting the O_2_ supply from tumors to these metabolically active tissues. This in turn will activate the HIF-1 cascade in the tumor in order to preserve the local homeostasis, which possibly leads towards angiogenesis [114, 115]. Similarly, increased orthotopic breast tumor perfusion without affecting tumor oxygenation has been observed after forced daily treadmill running [116]. In order to investigate the influence of this altered microenvironment on chemotherapy efficacy, the group of Jones et al. conducted another study in two different murine orthotopic breast cancer models using the same study protocol. As opposed to the authors' previous data, tumor hypoxia, now assessed with EF5 immunohistochemistry, was significantly lower in the exercise groups as compared to controls. MVD and vessel maturity were significantly higher in the exercised animals, which led to increases in tumor perfusion as observed before [117]. Importantly, these results were in line with other preclinical data obtained in a rat orthotopic prostate cancer model [118].

Voluntary wheel running as used in the mouse studies described above [114, 115, 117], may be an animal-friendly, stressless way to influence tumor oxygenation. Moreover, as it is sufficient to provide running wheels in the animals' cages, this method is relatively cheap and uncomplicated since it does not require sophisticated technical equipment. Another major advantage is that low intensity exercise does not influence tumor growth [114, 115, 117]. Nevertheless, results may seem contradictory. In the studies where increases in HIF-1 were observed, hypoxia as such was not quantified [114, 115]. Importantly, the upregulation of HIF-1*α* can also be the result of other mechanisms than a hypoxic microenvironment. The observed changes in perfusion [114, 115] on the other hand are most probably acute phenomena, whereas the hypoxia marker EF5 [117] primarily stains chronic hypoxia. Also, the site of inoculation should always be taken into consideration. As already discussed in Section 2.2.1, the subcutaneous space and its adjacent tissues are poorly perfused, which may explain at least partially why subcutaneously implanted tumors experience acute hypoxia during exercise [119]. More cell lines and more models should be studied in order to fully explore the high potential of exercise to create a differential hypoxic tumor model.

#### 2.3.5. Other Approaches

Other, less-studied external interventions to decrease tumor hypoxia include electrical stimulation [120] and the addition of Matrigel during tumor inoculations [121]. By studying the effect of electrical stimulation of the sciatic nerve in intramuscularly implanted liver and rhabdomyosarcoma tumors, significant increases in tumor pO_2_ and tumor blood flow were observed in both models, accompanied by decreases in the O_2_ consumption rate [120]. However, seeing its invasiveness and technical complexity, this technique may be less applicable, especially in longitudinal therapy response evaluation studies. In line with this, the use of Matrigel is also not supported, as no differences in hypoxia could be observed between tumors originating from FaDu cells in medium with or without Matrigel, respectively [121].

### 2.4. Pharmacological Interventions

Last decades, the search for radiosensitizers and hypoxia-targeting therapies has led to the development of a variety of promising pharmacological interventions to overcome tumor hypoxia. For an excellent recent review of this matter, we refer to Horsman and Overgaard [110]. Some examples of these therapeutics with potential usefulness for the creation of differential hypoxia are described below.

In order to increase O_2_ delivery to tumors, administration of erythropoietin (EPO), a substance naturally produced by the body, seems evident. For instance, in glioblastoma and carboplatin-treated NSCLC xenograft tumors, EPO administration resulted in significantly lower hypoxia as assessed polarographically or with HIF-1*α* immunofluorescence, respectively [122, 123]. However, EPO also improved muscle oxygenation [122], which potentially complicates hypoxia tracer TBR calculations (cf Section 2.3.1). Since EPO also acts as a stimulatory growth factor, it may have detrimental tumor effects via processes that are not hypoxia-related [110].

A more promising drug may be metformin, a relatively safe antidiabetic with a favorable pharmacokinetic profile. It could be argued that the administration of metformin to laboratory animals may mimic the clinical situation to a certain extent, as the drug is widely prescribed to diabetes patients. However, it should be emphasized that in most preclinical experiments, the drug is administered to nondiabetic mice, often using doses exceeding those considered safe in humans which may impair direct translation. Among different anticancer effects attributed to this drug, metformin is supposed to improve tumor oxygenation via its inhibitory effect on complex I of the mitochondrial electron transport chain [124]. Zannella et al. showed in HCT116 CRC models that administration of a single dose of metformin indeed decreased tumor hypoxia and consequently improved radiotherapy response using [^18^F]FAZA PET [125], results that we were able to confirm in a comparable set-up using [^18^F]HX4 PET in A549 NSCLC xenografts [126]. These observations are supported by similar observations obtained in xenograft models of prostate cancer and lung carcinoma using another inhibitor of the mitochondrial complex I, BAY 87-2243; [^18^F]FAZA uptake was significantly reduced after administration of this novel drug [86]. In FaDu xenografts, no effect of a single dose of metformin on EF5 staining was observed, but treatment for seven consecutive days caused a nonsignificant decrease in tumor hypoxia [127]. Despite these observations, the way in which metformin influences radiation response is not fully understood and may not be fully attributable to hypoxia-related phenomena [128]. Moreover, in highly hypoxic pediatric sarcoma models, it was shown that metformin had no additive value to chemotherapy response, as opposed to tumors with a better oxygenation state [129].

Another way to influence tumor oxygenation is the use of vascular-targeting agents. A well-studied example of such drugs is hydralazine, a vasodilator that relaxes arterial smooth muscle. At high doses, hydralazine decreases tumor perfusion through the “steal” phenomenon and thus increases tumor hypoxia [79]. For instance, uptake of the hypoxia tracer Prognox™ ([^99m^Tc]HL91) was increased after hydralazine administration in NSCLC and CRC xenograft models when compared to controls [130, 131]. Similarly, drops in pO_2_ were observed after hydralazine administration in CRC models [132]. However, the use of hydralazine to create a hypoxic model for investigating the cytotoxicity of the hypoxic prodrug AQ4N produced conflicting results [133, 134]. It was therefore suggested that in some tumor models, the effect of hydralazine may be too limited and too short-lived [134]. Other vascular-targeting agents include vascular disrupting agents [110], such as the novel combretastatin analogue OXi-4503, a tubulin-binding agent that selectively targets endothelial cells and damages existing tumor vessels [135, 136]. However, administration of such vascular-targeting drugs may lead to severe reductions in blood flow and thus ischemic cell death. This may in turn lead to difficulties in interpreting longitudinal PET imaging results as radiotracer delivery may be severely hampered. Another category includes the angiogenesis inhibiting agents [110]. It is generally believed that administration of angiogenesis inhibitors in a correct dose leads to vascular normalization and consequent decreases in tumor hypoxia [137], as shown with [^18^F]FMISO PET in two patient-derived pancreas xenograft models after dovitinib treatment [138]. However, also increases in [^18^F]FMISO after antiangiogenesis treatment have been reported [139]. Moreover, for all vascular targeting agents, timing of administration in combination with other therapies, mainly radiotherapy, is still controversial. It is supposed that vascular disrupting agents should be administered shortly after irradiation in order to be additive [110], which makes them unsuitable for the creation of differential hypoxic models.

Yet another drug-based approach is the use of agents that specifically target hypoxic cells. A first class includes O_2_ mimetics such as nimorazole. In Denmark, this drug is incorporated in routine radiotherapy treatment of head and neck cancer [119]. A second class includes hypoxic prodrugs such as TH-302 [110]. For instance, it was shown that TH-302 significantly decreased the hypoxic fraction, as assessed with [^18^F]HX4 PET, in rhabdomyosarcoma and NSCLC xenograft tumors. However, administration of TH-302 for five consecutive days also resulted in a significant growth delay as compared to control tumors [77], which limits its potential as an exclusive hypoxia modulator.

Despite the promising potential of some of the therapies discussed above as a radiosensitizer, pharmacological intervention may not be the optimal choice for the creation of a differential hypoxic model. Indeed, drugs may disturb the physiological state of the tumor or alter the clearance properties of the tracer [3] and may moreover give rise to complex drug interactions when performing therapy response evaluation studies.

## 3. Conclusions

In an ideal world, one would be able to detect inherently differential hypoxia within a single cohort of tumor-bearing laboratory animals. However, external manipulations may be indispensable, especially for the investigation and validation of novel hypoxia-targeting therapies. In this article, we reviewed a substantial number of promising techniques with the potential to alter tumor oxygenation in a preclinical in vivo setting. Obviously, none of these models will accurately mimic the complexity of human disease. Indeed, each individual discussed technique entails specific practical or ethical drawbacks and is subject to the influence of the other parameters. For instance, when creating a differential hypoxia model using the breathing approach, one should also take into consideration that the studied cell line, the range of tumor volumes, the food type, and fasting periods, body temperature, or anesthesia during imaging studies may all have a substantial influence on the degree of tumor hypoxia, or hypoxia PET tracer uptake, independently from the administered breathing gasses. Therefore, in theory, all of these factors should be monitored very strictly in order to prevent experimental disturbances. Tumor hypoxia is such a transient, complex, and very sensitive process, as a result of which it is extremely susceptible to a lot of internal and external influencing factors.

Indeed, the smallest disturbance in one of the discussed variables mentioned above may individually or in combination lead to unpredictable, unexpected, unreliable, or unreproducible experimental outcomes. Nevertheless, application in a clinical setting is not free of influence from external factors. In this respect, the use of mouse models offers important advantages over clinical research, such as the ease of biopsy specimen collection, the nondependence on laborious patient recruitment and not running the risk of failing to reach target goals, and the curtailment of patient heterogeneity, among others.

Mouse models have actually proven their usefulness, as, for instance, [^18^F]FMISO has successfully been implemented in patients after extensive preclinical in vivo validation. Validation of other hypoxia tracers is however still evolving. In this regard, this review provides a comprehensive overview and a better understanding of the applicable in vivo hypoxia modulation methods, but it also reveals that experimental modulation of tumor oxygenation remains a challenge. All of the reviewed methods may serve for specific experimental designs or hypotheses and in this way, they undoubtedly all contribute to the ongoing search for new biomarkers and cures for cancer.
